# Genomic characterization of *Staphylococcus aureus* isolated from patients admitted to intensive care units of a tertiary care hospital: epidemiological risk of nasal carriage of virulent clone during admission

**DOI:** 10.1128/spectrum.02950-23

**Published:** 2024-05-06

**Authors:** Takahiro Inagawa, Junzo Hisatsune, Shoko Kutsuno, Yasuhisa Iwao, Yumiko Koba, Seiya Kashiyama, Kohei Ota, Nobuaki Shime, Motoyuki Sugai

**Affiliations:** 1 Department of Emergency and Critical Care Medicine, Graduate School of Biomedical and Health Sciences, Hiroshima University, Minami-ku, Hiroshima, Japan; 2 Antimicrobial Resistance Research Center, National Institute of Infectious Diseases, Tokyo, Japan; 3 Department of Antimicrobial Resistance, Hiroshima University Graduate School of Biomedical and Health Sciences, Hiroshima, Japan; 4 Section of Clinical Laboratory, Division of Clinical Support, Hiroshima University Hospital, Hiroshima, Japan; 5 Division of Laboratory Medicine, Hiroshima University Hospital, Hiroshima, Japan; Icahn School of Medicine at Mount Sinai, New York, New York, USA

**Keywords:** ICU, *Staphylococcus aureus*, genomic sequence, ARGs, VFGs

## Abstract

We conducted a molecular epidemiological study of *Staphylococcus aureus* using whole-genome sequence data and clinical data of isolates from nasal swabs of patients admitted to the intensive care unit (ICU) of Hiroshima University hospital. The relationship between isolate genotypes and virulence factors, particularly for isolates that caused infectious diseases during ICU admission was compared with those that did not. The nasal carriage rates of methicillin-resistant *S. aureus* (MRSA) and methicillin-susceptible *S. aureus* (MSSA) in patients admitted to the ICU were 7.0% and 20.1%, respectively. The carriage rate of community-acquired (CA)-MRSA was 2.3%, accounting for 32.8% of all MRSA isolates. Whole-genome sequencing analysis of the MRSA isolates indicated that most, including CA-MRSA and healthcare-associated (HA)-MRSA, belonged to clonal complex (CC) 8 [sequence type (ST) 8] and SCC*mec* type IV. Furthermore, results for three disease foci (pneumonia, skin and soft tissue infection, and deep abscess) and the assessment of virulence factor genes associated with disease conditions [bacteremia, acute respiratory distress syndrome (ARDS), disseminated intravascular coagulopathy (DIC), and septic shock] suggested that nasal colonization of *S. aureus* clones could represent a risk for patients within the ICU. Particularly, MRSA/J and MSSA/J may be more likely to cause deep abscess infection; ST764 may cause ventilation-associated pneumonia, hospital-acquired pneumonia and subsequent bacteremia, and ARDS, and *tst-1*-positive isolates may cause DIC onset.

**IMPORTANCE:**

Nasal colonization of MRSA in patients admitted to the intensive care unit (ICU) may predict the development of MRSA infections. However, no bacteriological data are available to perform risk assessments for *Staphylococcus aureus* infection onset. In this single-center 2-year genomic surveillance study, we analyzed all *S. aureus* isolates from nasal swabs of patients admitted to the ICU and those from the blood or lesions of in-patients who developed infectious diseases in the ICU. Furthermore, we identified the virulent clones responsible for causing infectious diseases in the ICU. Herein, we report several virulent clones present in the nares that are predictive of invasive infections. This information may facilitate the design of preemptive strategies to identify and eradicate virulent MRSA strains, reducing nosocomial infections within the ICU.

## INTRODUCTION

Methicillin-resistant *Staphylococcus aureus* (MRSA) is often associated with invasive infections, such as ventilator-associated pneumonia (VAP), catheter-related bloodstream infection (CRBSI), surgical site infection (SSI), and wound infection, in trauma patients admitted to emergency rooms (ER) or intensive care units (ICU) (1). In Japan, the mortality rates of sepsis caused by *S. aureus* and MRSA were 21% and 25% in 2017, respectively, with death tolls of 17,157 and 4,224, respectively (2). Furthermore, 18%–31% of VAP and 10%–25% of CRBSI cases in Japan are caused by *S. aureus* (1). Hence, the prevalence of MRSA nasal colonization in patients admitted to the ICU may predict MRSA infections in the ICU (3, 4). Indeed, active MRSA surveillance increases the chances of contact precautions and decreases empirical treatment (4, 5). Moreover, early intervention for MRSA colonization using mupirocin may reduce the burden of severe MRSA infection; however, this may also induce mupirocin resistance in colonized *S. aureus* (6–8).

General intervention strategies are associated with significant economic costs to hospitals and patients. Therefore, screening for highly virulent clones and selective decolonization may contribute to the control of severe infection onset and diminish the risk of emerging antimicrobial resistance.

*S. aureus* displays diverse genotypes, and the genotypes of major clones differ between countries (9–12). Previous studies have indicated that regional prevalence of clones is changing (9, 13, 14); thus, molecular epidemiology studies may be useful for risk assessment, treatment selection, and information update. Few large-scale epidemiological studies on *S. aureus* infection in ICU are available, and no such study has been conducted using whole-genome sequencing data (10–13).

We conducted a molecular epidemiological study using the whole-genome sequences of isolates collected via nasal screening patients admitted to the ICU in a teaching hospital. To highlight high-risk clones in the ICU, we compared the relationship between genotypes and virulence determinants of isolates causing infectious diseases and those from in-patients without infectious diseases.

## RESULTS

### Isolation of *S. aureus*

**Fig 1:**
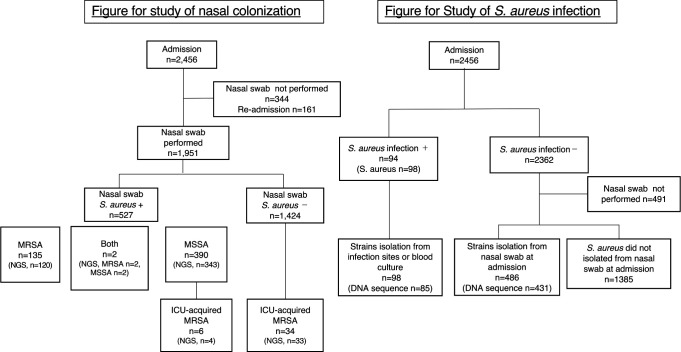
Schematic diagram of *S. aureus* isolation.

A total of 2,456 patients were admitted to ICU of Hiroshima University Hospital between 1 June 2017 and 31 May 2019 (Fig. 1). Of these patients, 505 (20.6%) were excluded from the study, 161 (6.6%) were readmitted, and 344 (14.0%) did not undergo nasal swabbing due to discharge or death within 48 h of admission.

Isolates from 1,951 nasal swabs collected from patients during their first visit were used in this study (Table S1). Of these isolates, 527 (27%), 392 (20.1%), and 137 (7.0%) were identified as *S. aureus*, methicillin-susceptible *S. aureus* (MSSA), and MRSA, respectively. Among the 137 MRSA isolates, 45 (32.8 %) and 92 (67.2 %) were identified as community-acquired (CA)-MRSA and healthcare-associated (HA)-MRSA, respectively. Furthermore, 40 (2.2%) patients acquired MRSA during their stay (after 24 h of admission) in the ICU, among which 6 were positive for MSSA and 34 were negative for *S. aureus* upon admission. These 40 isolates were designated as ICU-acquired MRSA.

In addition, 92/2,456 patients (3.8%) tested positive for *S. aureus* infection upon or after admission, and 98 isolates from lesions or blood cultures were identified as *S. aureus*. Among the 2,362 patients who tested negative for *S. aureus* infection, 486 (20.6%) were positive for *S. aureus* using nasal swabs and were defined as having non-infectious *S. aureus*.

### Molecular characterization of *S. aureus* isolated from different sources

Whole-genome sequencing analysis was conducted for 583 *S*. *aureus* isolates; 467 were obtained from initial nasal swabs upon admission (122 MRSA and 345 MSSA). Of the remaining 116 specimens, 85 were collected from infection sites (34 MRSA and 51 MSSA), of which 37 were ICU-acquired MRSA and 6 were ICU-acquired MRSA from the infection site. Of the initial nasal specimens, 431 were collected from patients who did not present with *S. aureus* infection (asymptomatic) during their ICU stay (107 MRSA and 324 MSSA). Multilocus sequence typing (MLST) and SCC*mec* typing were performed using the corresponding sequencing data.

**Fig 2:**
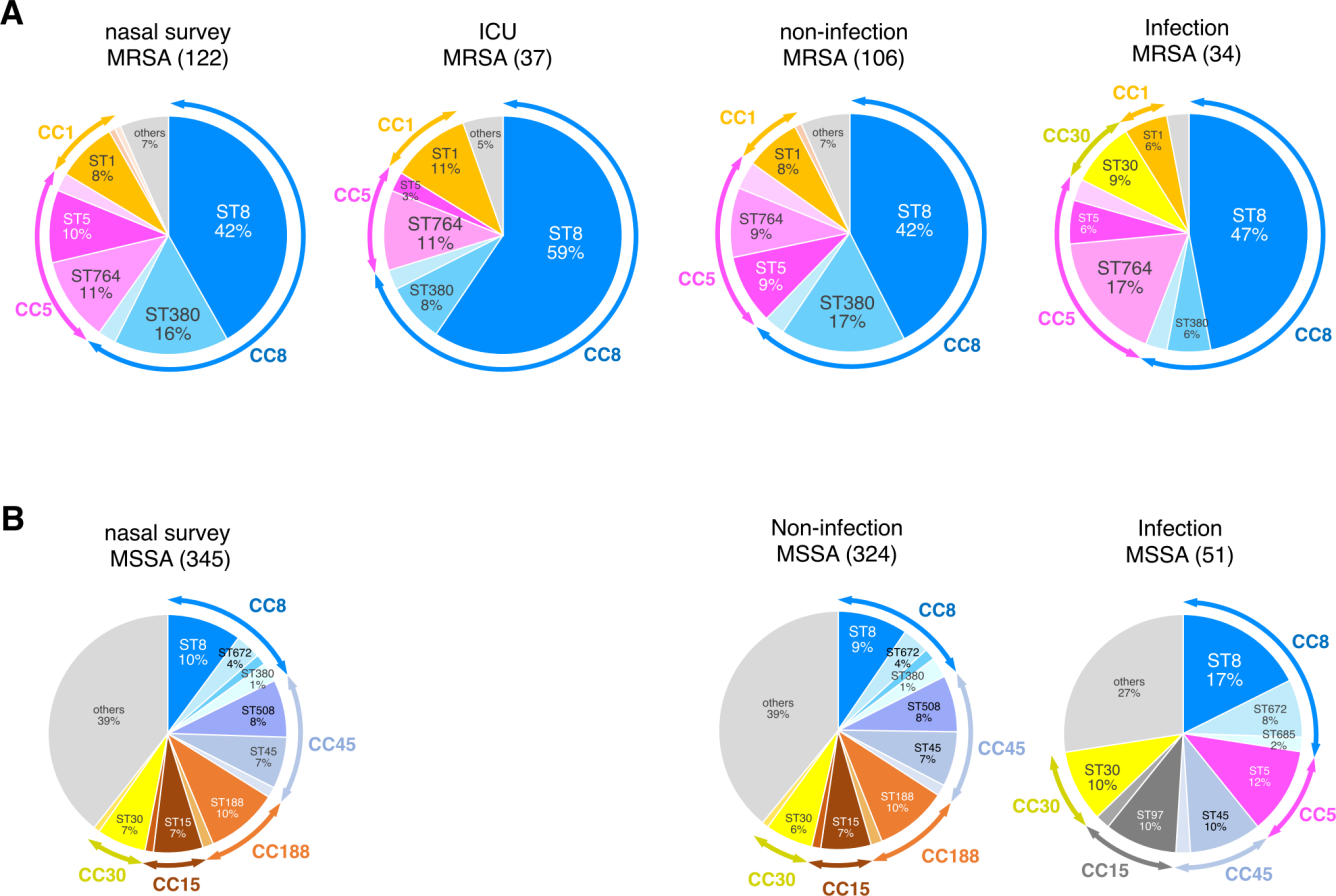
Proportion of STs of (**A**) MRSA and (**B**) MSSA isolates in each category. Arrows at both ends of the outer ring indicate CCs.

MLST revealed that most isolates from the initial screening belonged to clonal complexes (CC) 8 (73, 59.8%) and 5 (29, 23.7%), accounting for 84.4% of the total isolates (Fig. 2A). Among the CC8 isolates, Sequence Type (ST) 8 was the most abundant (51/73, 69.9%), followed by ST380 (19/73, 26.0%). Among the CC5 isolates, ST764 (14/29, 48.3%) and ST5 (12/29, 41.4%) were abundant. The CC and ST patterns of MRSA isolated from nasal swabs of patients who did not develop *S. aureus* infection during their ICU stay (non-infectious MRSA) were similar to those from the initial screening. In addition, CC8 (26, 70.3%) was the most prevalent in isolates from nasal swabs during the ICU stay, followed by CC5 (15, 13.5%) and CC1 (14, 10.8%); however, the prevalence of ST8 was much higher (22, 59.5%) than from initial nasal swab screening (51, 41.8%) or from the infection site (16, 47.1%). Similar observations were recorded for MRSA isolates from the lesions or blood of patients who developed *S. aureus* infection; however, CC30 (ST30) (3, 8.8%) was more prevalent than CC1 (2, 5.9%). Furthermore, 17.6% of the total isolates was ST764, which was much higher than that of the other isolates.

**Fig 3:**
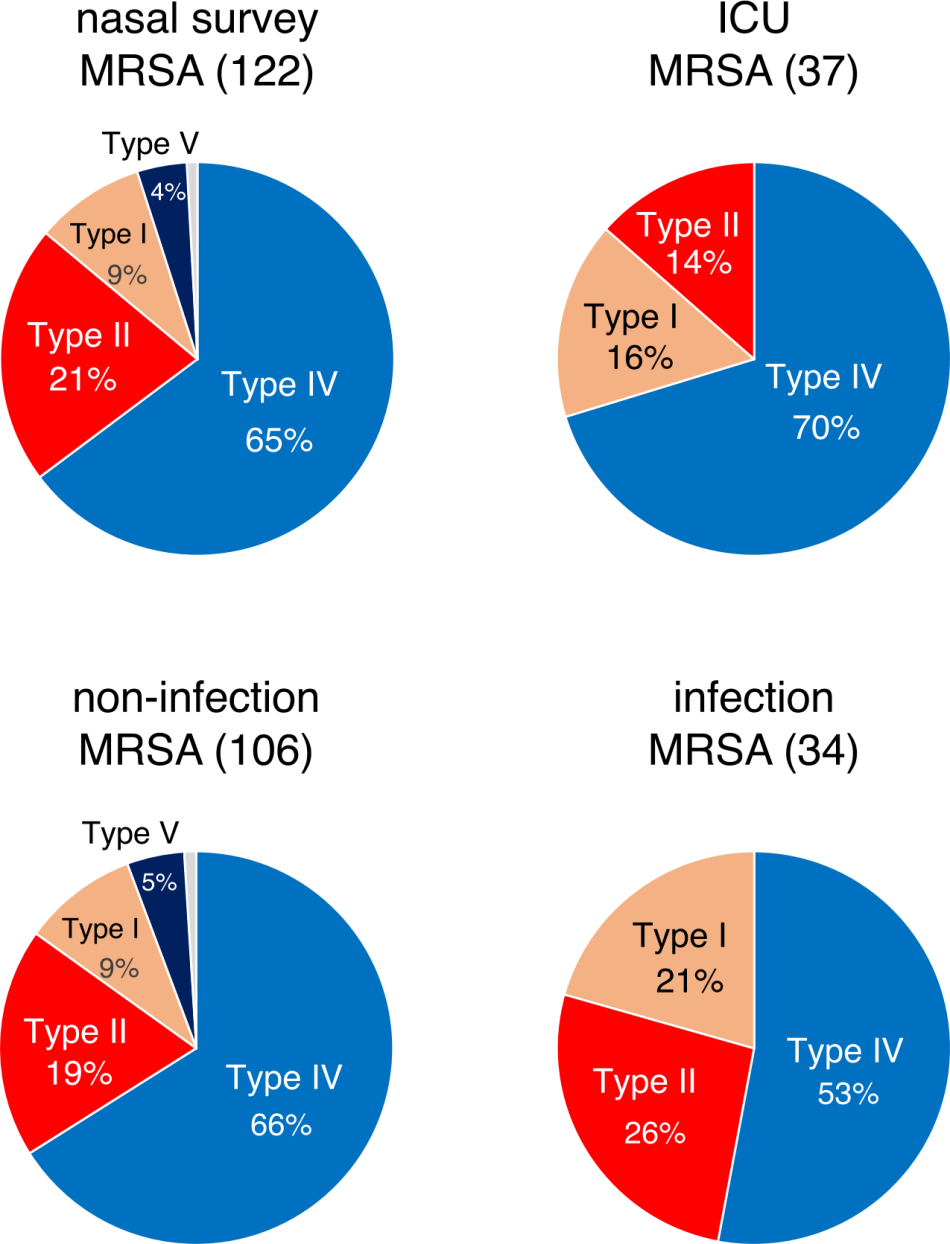
Proportion of SCC*mec* types in each category.

SCC*mec* typing revealed that MRSA isolates from the initial screening included SCC*mec*IV (79/122, 64.8%), SCC*mec*II (26/122, 21.3%), and SCC*mec*I (11/122, 9.0%), reflecting the dominant CC8 lineage (Fig. 2A and 3). These findings were similar to those of non-infectious MRSA isolates (Fig. 3). An increased ratio of SCC*mec*I MRSA (6/37, 16%) was observed from nasal swabs of patients during their ICU stay, whereas those isolated from the site of infections displayed different patterns: SCC*mec*IV (52.9%, 18/34), SCC*mec*II (26.5%, 9/34), and SCC*mec*I (20.6%, 7/34).

The combination of MLST and SCC*mec* typing revealed the proportion of specific clones. The proportions were the following: ST1-MRSA-SCC*mec*IV 8.2% (10/122), ST2725-MRSA-SCC*mec*IV 0.8% (1/122), ST5-MRSA-SCC*mec*II [New York/Japan (9 )] 6.6% (8/122), ST764-MRSA-SCC*mec*II 11.5% (14/122), and ST8-MRSA-SCC*mec*IVl [MRSA/J (15)] 15.6% (19/122) (Table S3). On the other hand, ST8-MRSA-SCC*mec*IVa-ACME [USA300 (15)] was not detected in this study.

MLST of MSSA isolates from the initial screening of nasal swabs and patients who did not experience *S. aureus* infection during their ICU stay revealed various sequence types, including CC8, CC45, CC188, CC15, and CC30 (Fig. 2B). In contrast, the MLST patterns of MSSA isolates from the lesions or blood of patients with infectious MSSA included CC8 (27.4%, 14), CC5 (11.8%, 6), CC45 (11.8%, 6), CC15 (11.8%, 6), and CC30 (9.8%, 5). An increased ratio of CC8 (infection 27.5%, initial nasal swab 17.7%) and CC5 (infection 11.8%, initial nasal swab 5.5%) was observed. Overall, ST380, ST764, and ST1 were unique to MRSA, while ST508, ST45, ST188, and ST15 were unique to MSSA (Fig. 2A and B).

**Fig 4:**
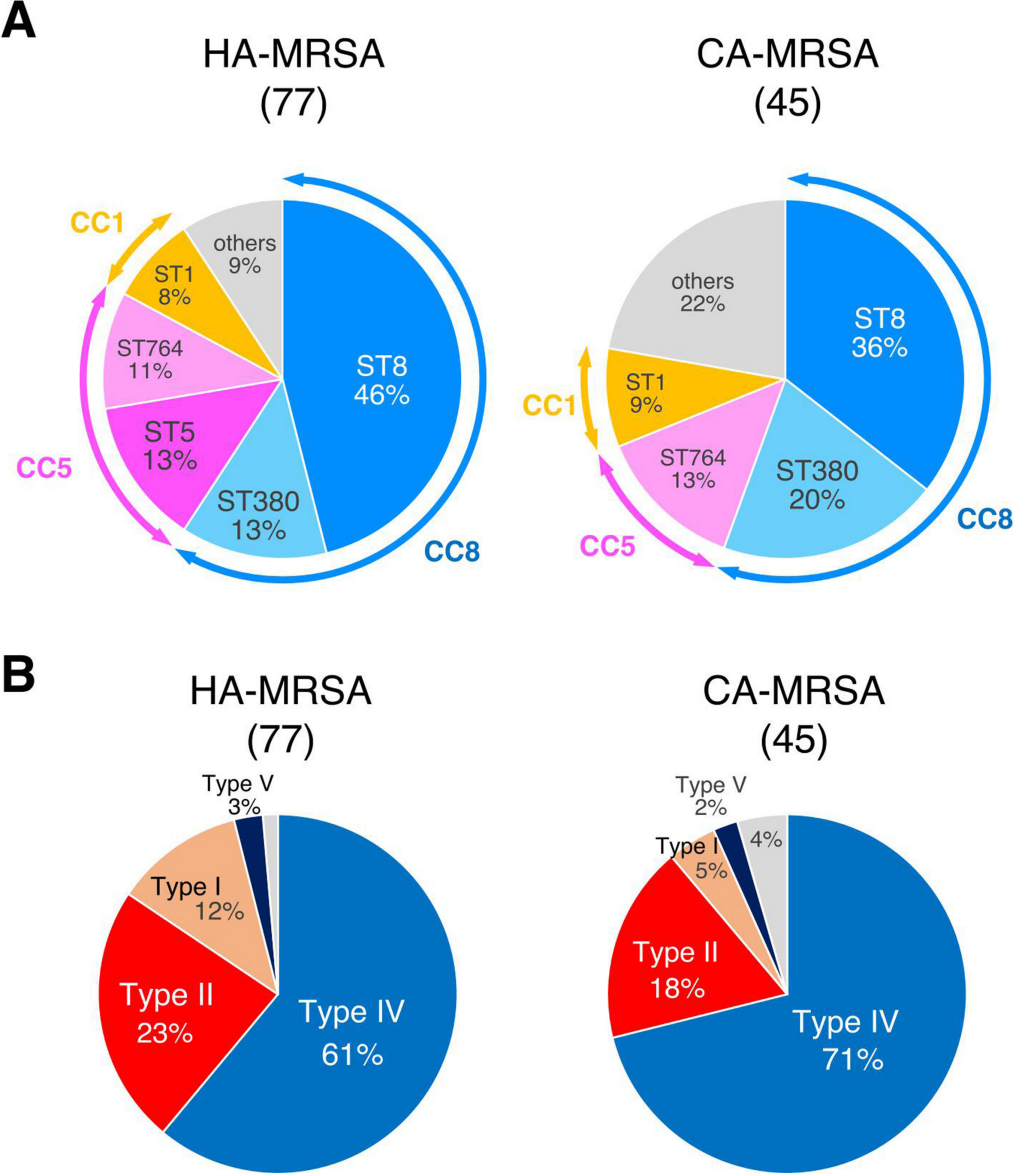
Proportion of (**A**) STs and (**B**) SCC*mec* types in HA-MRSA and CA-MRSA.

The clinical classification of MRSA indicated that 55.6% (25/45) of CA-MRSA and 58.4% (45/77) of HA-MRSA belonged to CC8, while 17.8% (8/45) and 23.3% (18/77) belonged to CC5, respectively (Fig. 4). Notably, ST5 was observed only in HA-MRSA isolates. Most SCC*mec* types were SCC*mec*IV in CA-MRSA (32/45, 71.1%) and HA-MRSA (47/77, 61.0%) isolates.

### Prevalence of VFGs and ARGs in *S. aureus* isolated from nasal swabs and infection sites

**Fig 5:**
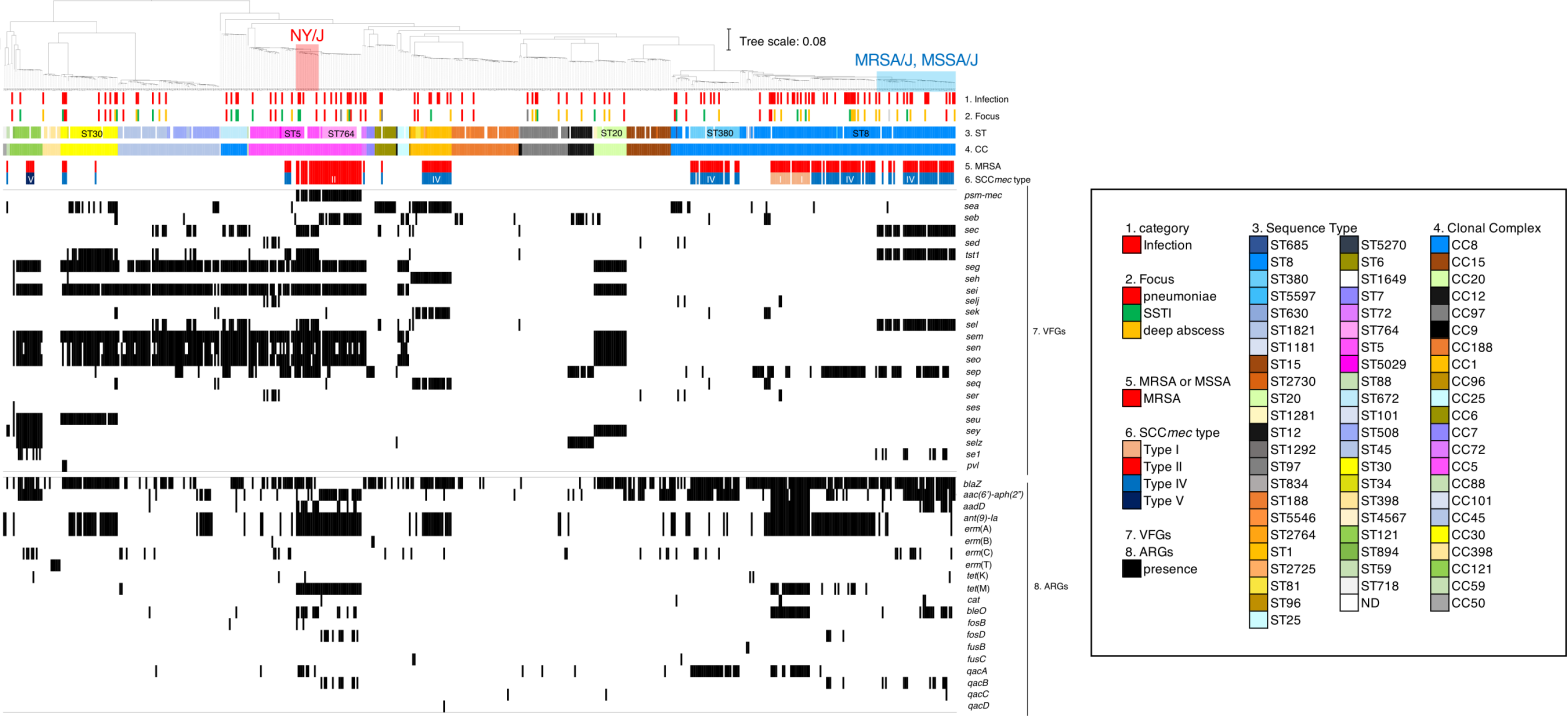
Pan-genome single nucleotide polymorphism (SNP)-based phylogenetic analysis of 583 *S*. *aureus* isolates in this study. A parsimony tree was constructed based on the pan-genome 249,203 SNP sites with branch lengths expressed in terms of changes per number of SNPs using kSNP3.021. Heatmap: column 1, presence or absence of infection; column 2, disease focus such as pneumoniae, SSTI, and deep abscess; column 3, each color represents an ST; column 4, each color represents a CC; column 5, methicillin-resistant *S. aureus* (MRSA; red); column 6, SCC*mec* types of MRSA; columns 7 and 8, the presence/absence of VFGs and ARGs, with black representing the presence of genes. MRSA/J is an MRSA clone belonging to CC8 (ST8), and SCC*mec* type is IVl and possesses *tst-1*. MSSA/J is an MSSA clone belonging to CC8 (ST8) and possesses *tst-1*. New York/Japan is an MRSA clone belonging to CC5 (ST5), and SCC*mec* type is II and possesses *tst-1*.

**Fig 6:**
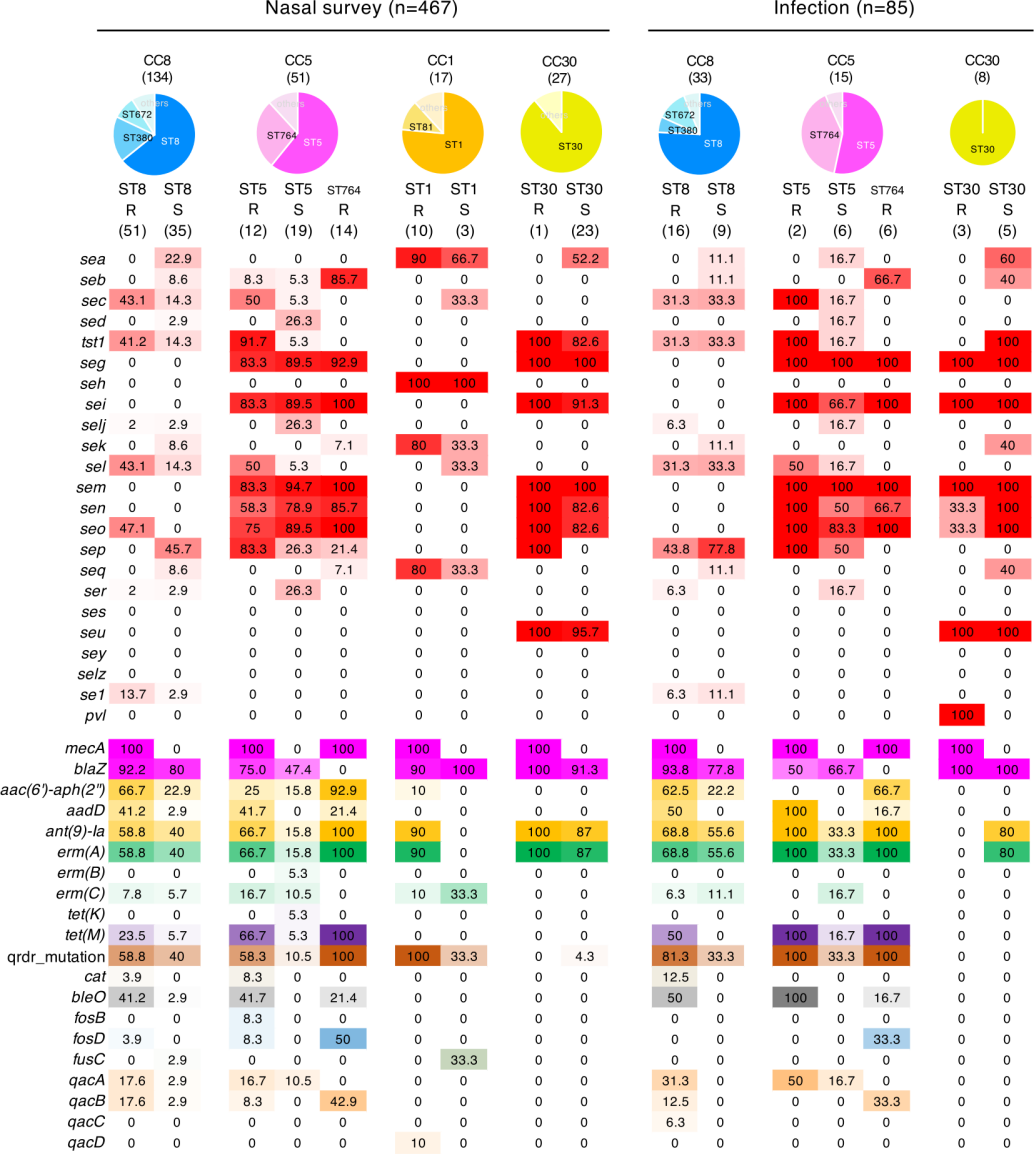
Characteristics of VFG or ARG patterns of representative STs of *S. aureus* isolates on nasal survey and infection groups. The proportion of several VFGs and ARGs of each ST is presented using heatmaps. The numbers in the heatmap columns are shown as percentages. R, MRSA; S, MSSA.

A whole-genome-based phylogeny of 583 *S*. *aureus* draft genomes was reconstructed using kSNP3.0 to characterize the population structure of ICU isolates with the metadata for infection foci, ST, CC, virulence factor genes (VFGs), and antimicrobial-resistant genes (ARGs) (Fig. 5). The prevalence of VFGs and ARGs in major STs isolated from nasal swabs and infection sites was also explored (Fig. 6).

Approximately 40% of ST8 MRSA isolates from nasal swabs harbored the enterotoxin gene sets *sec*, *tst-1*, *sel*, and *sep*, while 13.7% harbored *se1* (Fig. 5 and 6). In contrast, ST8 MSSA from nasal swabs harbored *sea* (9/35, 25.7%), *seb* (3/35, 8.6%), and *sep* (18/35, 51.5%), while 20.0% possessed the gene sets *sec*, *tst-1*, and *sel* (Fig. 6). The VFG sets of ST8 MRSA and ST8 MSSA from the infection site were similar, and approximately 30% possessed a common gene set (*sec*, *tst-1*, and *sel*). The ratios of *sep* in ST8 MRSA and ST8 MSSA was 44% and 78%, respectively. A cluster of ST8 carrying *sec*, *tst-1*, *sel*, and *sep* represents the MRSA/J lineage (ST8-MRSA-SCC*mec*IVl, *tst-1* positive) (15) and its methicillin-susceptible counterpart (MSSA/J) (Fig. 5). In addition, 94.7% (18/19) of MRSA/J was positive for *aac(6*′)-*aph(2*′′). Draft sequence data indicated that 37.3% (19/51) of ST8 MRSA isolates from the nasal swab was MRSA/J (Table 1). In contrast, ST8 did not include the USA300 lineage (ST8-SCC*mec*IVa, *pvl* positive).

**TABLE 1:** Proportion of MRSA/J or MSSA/J in ST8 each category^*a*^

	MRSA/J in ST8-MRSA	MSSA/J in ST8-MSSA
Nasal survey	19/51 (37.3)	7/35 (20)
ICU	6/22 (27.3)	–^*b*^
Non-infection	16/45 (35.6)	7/31 (22.6)
Infection	4/14 (28.6)	3/9 (33.3)

^*a*^ Numbers in brackets indicate percentages.

^*b*^ Not detected.

The common gene sets of ST5 MRSA from the initial nasal swab and the infection site were *sec*, *tst-1*, *seg*, *sei*, *sel*, *sem*, *sen*, *seo*, and *sep* (Fig. 6). The positivity of the gene set was 50%–90% in ST5 MRSA from the initial nasal swab and 100% in ST5 MRSA from the infection site, except *sel* (Fig. 6). A similar gene set was present in ST5 MSSA from the initial nasal swab and infection site; however, the positive ratio of *tst-1* was notably lower than that of ST5 MRSA (5% vs. 92% in the initial nasal swab and 17% vs. 100% in the infection site). New York/Japan clone (ST5-SCC*mec*II) accounted for only 6.6% (8/122) of initial MRSA nasal carriage (Table S3), but it accounted for 66.7% of ST5 (8/12) (Fig. 5). The ST764 isolates were all MRSA, and most possessed a common set of virulence genes, including *psm-mec*, *seb*, *seg*, *sei*, *sem*, *sen*, and *seo* (Fig. 5 and 6). All ST764 and ST5-SCC*mec*II MRSA (New York/Japan clone) isolates were positive for *ant(9)-Ia*, *erm*(A), and *tet*(M). Some ST764 isolates also possessed *fosD* and *qacB*. The common gene sets of ST30 MRSA and ST30 MSSA from the initial nasal swab and infection site were *seg*, *sei*, *sem*, *sen*, *seo*, and *seu* (Fig. 5 and 6). Most of the ST30 MSSA isolates from the infection site possessed *tst-1*, and 100% of the ST30 MRSA isolates from the infection site possessed *pvl* although the total number was small (Fig. 6). MRSA isolates of major STs, except ST30, possessed a significantly higher ratio of aminoglycoside resistance genes, *erm*(A) and *tet*(M), suggesting multiple resistance (Fig. 5 and 6). A higher ratio of *qacA* positivity was also observed in ST380 MRSA and ST8 SCC*mec*I MRSA (Fig. 5).

### Characteristics of *S. aureus* isolated from lesions or blood from patients who developed infectious diseases

**Fig 7:**
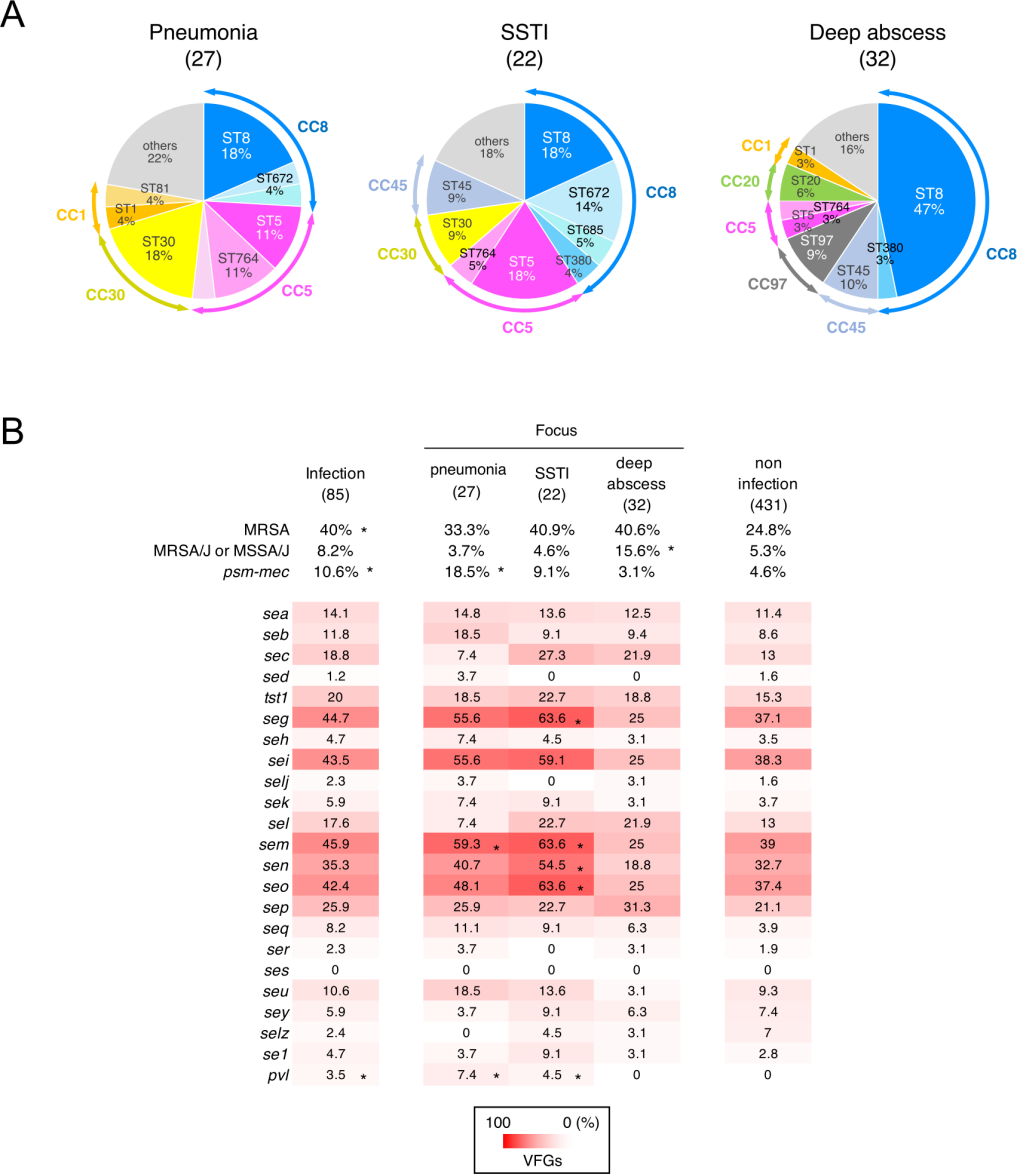
Characteristics of (**A**) ST and (**B**) VFG patterns of *S. aureus* isolates on pneumoniae, SSTI, and deep abscess. Arrows at both ends of the outer ring on (**A**) pie chart indicate CCs. (**B**) The numbers in the VFGs heatmap are shown as percentages.

Infectious diseases included pneumonia (27, 31.8%); skin and soft tissue infection (SSTI) (22, 25.9%); deep abscesses, including infectious endocarditis and multiple abscesses (32, 37.6%); device infection (3, 3.5%); and bacteremia with unknown foci (1, 1.2%) (Fig. 7B). Meanwhile, foci diseases caused by *S. aureus* included bacteremia (41, 48.2%) and acute respiratory distress syndrome (ARDS; 20, 23.5%), disseminated intravascular coagulation (DIC; 36, 42.4%), and septic shock (36, 42.4%) (Table 2). We compared the MLST results and virulence genes of the isolates from the three foci. In pneumonia, the isolates were classified as ST8 (18%), ST30 (18%), ST764 (11%), and ST5 (11%), of which the ST764 ratio was higher in isolates from pneumonia than in other foci (Fig. 7A). The SSTI isolates included ST8 (18%), ST5 (18%), ST672 (14%), ST30 (9%), and ST45 (9%). In deep abscesses, isolates included ST8 (47%), ST45 (10%), ST97 (9%), and ST20 (6%), in which the ratio of ST8 was the highest in the three foci, and ST20 was primarily found in isolates from abscesses (15.6%). Compared with those from nasal swabs of patients who did not present with *S. aureus* infection during their ICU stay, the isolates from infection foci showed higher rates of *mecA* and *psm-mec* (Fig. 7B). The positive ratios of *mecA* in isolates from patients with pneumonia, SSTI, and deep abscesses were 33.3% (9/27), 40.9% (9/22), and 40.6% (13/32), respectively. Among the three foci, the isolates from patients with pneumonia showed significantly higher ratios of *psm-mec*, *sem*, and *pvl*. In contrast, isolates from SSTI revealed a statistically higher ratio of *seg*, *sem*, *sen*, *seo*, and *pvl*. MRSA/J and MSSA/J were the most prevalent isolates from deep abscesses (15.6%).

**TABLE 2:** Prevalence of MRSA and VFGs in bacteremia, ARDS, DIC, and septic shock^*a*^

	Bacteremia			ARDS			DIC			Septic shock		
	+41	−44	*P* value	+20	−65	*P* value	+36	−49	*P* value	+36	−49	*P* value
MRSA VFGs	17 (41.5)	16 (36.4)	0.6298	12 (60)	21 (32.3)	0.0278	12 (33.3)	22 (44.9)	0.1775	14 (38.9)	19 (38.8)	0.9915
*psm-mec*	5 (12.2)	4 (9.1)	0.6421	5 (25)	4 (6.2)	0.0166	4 (11.1)	5 (10.2)	0.8932	4 (11.1)	5 (10.2)	0.8932
*sea*	3 (7.3)	8 (20.5)	0.0822	2 (10)	10 (15.4)	0.5453	5 (13.9)	7 (14.3)	0.9586	6 (16.7)	6 (12.2)	0.5648
*seb*	8 (19.5)	2 (4.6)	0.0324	4 (20)	6 (9.2)	0.1912	4 (11.1)	6 (12.2)	0.8726	6 (16.7)	4 (8.2)	0.2292
*sec*	6 (14.6)	10 (22.7)	0.3377	4 (20)	12 (18.5)	0.8777	7 (19.4)	9 (18.4)	0.9002	6 (16.7)	10 (20.4)	0.6613
*sed*	1 (2.4)	0 (0)	0.2974	1 (5)	0 (0)	0.0698	1 (2.8)	0 (0)	0.2406	1 (2.8)	0 (0)	0.2406
*tst1*	7 (17.1)	10 (22.7)	0.5138	4 (20)	13 (20)	1.0000	12 (33.3)	5 (10.2)	0.0084	9 (25)	8 (16.3)	0.3257
*seg*	18 (43.9)	20 (45.5)	0.8856	11 (55)	27 (41.5)	0.2908	18 (50)	20 (40.8)	0.4002	17 (47.2)	21 (42.9)	0.6893
*seh*	1 (2.4)	3 (6.8)	0.3408	1 (5)	3 (4.6)	0.9434	1 (2.8)	3 (6.1)	0.4718	2 (5.6)	2 (4.1)	0.7512
*sei*	17 (41.5)	20 (45.5)	0.7107	10 (50)	28 (43.1)	0.5057	17 (47.2)	20 (40.8)	0.5563	17 (47.2)	20 (30.8)	0.5563
*selj*	1 (2.4)	1 (2.3)	0.9597	1 (5)	1 (1.5)	0.3718	1 (2.8)	1 (2)	0.8247	1 (2.8)	1 (2)	0.8247
*sek*	3 (7.3)	2 (4.6)	0.5874	1 (5)	4 (6.2)	0.8479	3 (8.3)	2 (4.1)	0.4104	3 (8.3)	2 (4.1)	0.4104
*sel*	6 (14.6)	9 (20.5)	0.4803	4 (20)	11 (16.9)	0.7523	6 (16.7)	9 (18.4)	0.8386	5 (13.9)	10 (20.4)	0.4313
*sem*	18 (43.9)	21 (47.7)	0.7236	11 (55)	28 (43.1)	0.3500	18 (50)	21 (42.9)	0.5138	18 (50)	21 (42.9)	0.5138
*sen*	12 (29.3)	18 (40.9)	0.2605	9 (45)	21 (32.3)	0.3044	16 (44.4)	14 (28.6)	0.1310	12 (33.3)	18 (36.7)	0.7454
*seo*	15 (36.6)	21 (47.7)	0.2981	9 (45)	27 (41.5)	0.7845	17 (47.2)	19 (38.8)	0.4364	16 (44.4)	20 (43.5)	0.7381
*sep*	8 (19.5)	14 (31.8)	0.1930	3 (15)	19 (29.2)	0.2039	13 (36.1)	9 (18.4)	0.0660	7 (19.4)	15 (30.6)	0.2404
*seq*	3 (7.3)	4 (9.1)	0.7663	1 (5)	6 (9.2)	0.5473	3 (8.3)	4 (8.2)	0.9775	4 (11.1)	3 (6.1)	0.4084
*ser*	1 (2.4)	1 (2.3)	0.9597	1 (5)	1 (1.5)	0.3718	1 (2.8)	1 (2)	0.8247	1 (2.8)	1 (2)	0.8247
*ses*	0 (0)	0 (0)	–^*b*^	0 (0)	0 (0)	–	0 (0)	0 (0)	–	0 (0)	0 (0)	–
*seu*	4 (9.8)	5 (11.4)	0.8098	2 (10)	7 (10.8)	0.9221	5 (13.9)	4 (8.2)	0.3966	4 (11.1)	5 (10.2)	0.8932
*sey*	2 (4.9)	3 (6.8)	0.7040	1 (5)	4 (6.2)	0.8479	2 (5.6)	3 (6.1)	0.9126	3 (8.3)	2 (4.1)	0.4104
*selz*	0 (0)	2 (4.6)	0.1671	0 (0)	2 (3.1)	0.4273	0 (0)	2 (4.1)	0.2199	0 (0)	2 (4.1)	0.2199
*se1*	1 (2.4)	3 (6.8)	0.3408	0 (0)	4 (6.2)	0.2558	0 (0)	4 (8.2)	0.0791	1 (2.8)	3 (6.1)	0.4718
*eta*	0 (0)	1 (2.3)	0.3315	0 (0)	1 (1.5)	0.5768	0 (0)	1 (2)	0.3886	0 (0)	1 (2)	0.3886
*etb*	0 (0)	0 (0)	–	0 (0)	0 (0)	–	0 (0)	0 (0)	–	0 (0)	0 (0)	–
*etd*	0 (0)	0 (0)	–	0 (0)	0 (0)	–	0 (0)	0 (0)	–	0 (0)	0 (0)	–
*pvl*	2 (4.9)	1 (2.3)	0.5154	1 (5)	2 (3.1)	0.6836	0 (0)	3 (6.1)	0.1307	1 (2.8)	2 (4.1)	0.7475

^*a*^ Numbers in brackets indicate percentages.

^*b*^ Not detected.

Furthermore, we assessed virulence factor genes associated with infectious diseases caused by *S. aureus*. Bacteremia was significantly associated with isolates positive for *seb*, whereas ARDS was significantly associated with isolates positive for *mecA* and *psm-mec* (Table 2). In contrast, DIC was significantly associated with isolates positive for *tst-1*. However, no related virulence factor genes were detected during septic shock. Additionally, to identify variables that may influence each severe disease state, multivariate analysis was performed. Bacteremia was found to be independently associated with *seb*, DIC with *tst-1*, and ARDS with *psm-mec* (Table S2).

## DISCUSSION

This large-scale epidemiology study investigated the presence of *S. aureus* in the nasal cavity of more than 2,000 patients admitted to the ICU. The overall carriage rates of MRSA and MSSA were 7.0% and 20.1%, respectively. CA-MRSA accounted for 32.8% (45/137) of all MRSA isolates. MLST revealed that most MRSA isolates belonged to CC8, followed by CC5, suggesting a transition of the major CC in MRSA. Several small-scale domestic studies have reported an increase in CC8 (ST8) levels in MRSA (16–20). In parallel with the CC transition, the dominant SCC*mec* type changed from SCC*mec*II to SCC*mec*IV. Although the most predominant MRSA clone was previously CC5-ST5-SCC*mec*II (New York/Japan clone) (9), this clone accounted for only 6.6% of the MRSA in the current study (Table S3). Surveillance of a Japanese tertiary hospital in 2015 demonstrated that SCC*mec*II decreased from 90.0% to 74.3% (21). Meanwhile, in 2017, the proportions of SCC*mec*IV and SCC*mec*II in a tertiary care hospital were 52.3% and 28.5%, respectively (22), indicating an increase in SCC*mec*IV prevalence. The predominance of SCC*mec*IV was also observed in CA-MRSA, HA-MRSA, and MRSA isolated from nasal swabs of patients during their ICU stay (Fig. 3 and 4).

The Japan Nosocomial Infection Surveillance data indicate that the isolation rate of MRSA (computed as the number of patients with positive MRSA culture over the total number of patients from whom specimens were collected) was 6.4%–6.6% (2015–2019) (23), which was comparable to the rate obtained in this study. In comparison, Panayotis et al. (3) reported that the carriage rates of MRSA in ICU patients in South Europe, North/Central Europe, North America, Asia, and South America were 3.5%, 4.4%, 8.8%, 12%, and 13.1%, respectively, suggesting that the carriage rate of MRSA in this study is closer to that in North America or Europe than in Asia. Additionally, a systematic review and meta-analysis performed by Wong et al. (24) reported that the ratio of CA-MRSA to total MRSA in a hospital setting was 0.7%–10.4%; the ratio of CA-MRSA in the current study was within this range.

Previously, the classification of MRSA into CA-MRSA and HA-MRSA using clinical standards supported the prediction of the SCC*mec* type and characteristics of the isolate, such as virulence factor repertoire and antimicrobial resistance (25). However, the results of the present study indicated that CC8 was the predominant group in CA-MRSA, HA-MRSA, and ICU-acquired MRSA, with predominant SCC*mec* type IV (Fig. 4), suggesting that such a clinical classification will no longer provide further insights into the virulence of isolates based on clonal differences between CA-MRSA and HA-MRSA.

ST8 was the most prevalent ST among the isolates from the infection sites, particularly from deep abscesses (Fig. 7A). Of these isolates from the infection site, 28.6% was MRSA/J, an endemic clone that causes severe invasive infectious diseases (Table 1) (15, 26, 27). Meanwhile, MSSA/J accounted for approximately 33.3% of the ST8-MSSA isolates from the infection site. Hence, MRSA/J and/or MSSA/J were implicated in deep abscesses (Fig. 7B). Furthermore, these strains were isolated from nasal swabs of patients in the ICU, suggesting that nasal carriage of these clones could be a potential risk factor for severe invasive skin infections.

Most CC5 isolates belonged to ST5 or ST764 (Fig. 5). CC5 isolates carried the *egc* cluster (*seg*, *sei*, *sem*, *sen*, and *seo*) and were frequently isolated from pneumonia and SSTI foci. Among the ST5 clones isolated from the infection site, only two isolates belonged to the authentic New York/Japan clone (ST5-SCC*mec*II carrying *tst-1*). However, these two strains caused DIC, suggesting that the New York/Japan clone maintains high virulence. The ST764 was first reported as a variant of ST5 in a 2001–2005 study in Japan (28), and several subsequent reports have been published (16, 29, 30). In our study, the primary focus of the ST764 isolates was pneumonia (Fig. 7A). Similarly, in 2017, Kaseida et al. (15) reported that ST764 is associated with pneumonia. Moreover, the assessment of virulence factor genes with disease conditions in our study suggested that bacteremia was associated with *seb* (Table 2; Table S2). The major ST carrying *seb* identified in this study was ST764. In contrast, ARDS is associated with *mecA* and *psm-mec. psm-mec* was present only in the ST764 and New York/Japan clones (Fig. 5). These results suggest that nasal colonization by ST764 may be a significant threat to HAP/VAP and the subsequent cause of bacteremia and ARDS in the ICU.

CC30 (ST30) commonly carried the *egc* cluster, and most ST30-MRSA possessed *tst-1* (Fig. 5). Although only a few were detected, all ST30 MRSA isolates from the infection site possessed *pvl*. PVL is a cytolytic toxin composed of a protein complex of the *lukS-PV* and *lukF-PV* gene products and is as an etiological agent of necrotizing pneumonia (31). USA300 is a well-known CA-MRSA of ST8 carrying *pvl* (15, 30); however, in Japan, the MRSA-positive detection rate for *pvl* is only 0.7% (25). In this study, only three isolates were positive for *pvl* and all belonged to ST30; however, all were isolated from the infection site. ST380 is classified as CC8 and approximately 80% of ST380 cases are MRSA carrying SCC*mec*IV. ST380 MRSA was the second most common isolate detected during our initial screening of nasal swabs (Fig. 2). ST380 MRSA has only been reported in Japan, suggesting that it is an endemic clone (19, 32). ST380 MRSA carries *qacA*, a resistance gene for organic cationic compounds, such as benzalkonium chloride and chlorhexidine, which may pose a risk of spreading antiseptic resistance (Fig. 4) (33, 34). Although the frequency of isolation from the infection site was low, the high rate of nasal carriage raised concerns about environmental contamination and clonal spread (Fig. 2 and 7A).

This study had several limitations. First, this was a single-center study involving the ICU department and did not, therefore, reflect nationwide epidemiology. Second, 16.5% of enrollments dropped out, and only nasal swabs were used to screen for *S. aureus*. Third, we did not examine all factors associated with patient risk. Nevertheless, this is the first large-scale epidemiological study of *S. aureus* infection in an ICU in Japan. While previous molecular epidemiological studies have focused on isolates from the infection foci, we attempted to include all isolates detected via carriage screening upon patient admission in our molecular epidemiological studies, better reflecting the true epidemiology of *S. aureus* infection in the ICU department.

## MATERIALS AND METHODS

### Sample collection

*S. aureus* was isolated from clinical specimens collected from patients admitted to the ICU at Hiroshima University Hospital from 1 June 2017 to 31 May 2019. Nasal swabs were collected from hospitalized patients within 24 h of, and every week during, admission. For patients admitted several times, the isolates from the first visit were used. To characterize *S. aureus*-caused infectious diseases, *S. aureus* was isolated from blood cultures or lesions at the infection site of patients admitted to ICU. When several isolates were obtained from the same lesion or multiple blood cultures of the same patient, the first isolate was selected for analysis. *S. aureus* isolates from patients diagnosed with sepsis/septic shock, DIC, and ARDS were compared with those from other studies (35–37). For clinical classification, HA-MRSA was defined as MRSA isolated within 48 h of admission and fulfilling at least one of the following criteria: patient with previous MRSA-positive infection, admitted within the past year, stayed in a long-term care facility, and underwent surgery, hemodialysis, or indwelling catheter. Isolates from patients who tested negative for nasal swab upon admission but tested positive after 24 h were classified as ICU-acquired MRSA, while other isolates were defined as CA-MRSA (38, 39). *S. aureus* was identified following the standard protocol of the VITEK2 GP Identification Card (bioMerieux Japan, Tokyo).

### Genome sequence analysis

Genomic DNA purification, library preparation, Illumina sequencing, and bioinformatic analysis were performed as previously described (40). Sequencing was performed using an Illumina HiSeq X FIVE platform (Macrogen Japan Co., Japan). Raw reads were assembled using Shovill v1.0.9 (available at https://github.com/tseemann/shovill) with the default settings. MLST analysis was performed using mlst v2.19.0 (available at https://github.com/tseemann/mlst). ARGs or VFGs were searched using ResFinder (updated on 23 January 2022) or VFDB (updated on 13 April 2022), respectively, on Abricate v1.0.1. SCC*mec* typing using SCC*mec*Finfer (default settings) was performed at the Center for Genomic Epidemiology (http://www.genomicepidemiology.org/). DDBJ Sequence Read Archive data set for this study was deposited in DNA Data Bank of Japan (http://www.ddbj.nig.ac.jp/) under BioProject accession number PRJDB13532.

### Construction of phylogenetic tree

kSNP3.021 (41) was employed to identify the pan-genome SNPs of the 583 *S*. *aureus* isolates and estimate the parsimony tree based on these SNPs. kSNP3.021 algorithm identifies SNPs based on unique stretches of nucleotides present in all genomes having SNPs in their middle position. The optimal size of the nucleotide regions flanking the SNPs (*k*-mer) was identified using the program Kchooser available with the package. A *k*-mer size of 19 was applied to identify SNPs. The 249,203 SNP sites were extracted from the pan-genome alignment. The SNP matrix file (SNPs_all_matrix.fasta, DOI: 10.6084/m9.figshare.25526326) generated by this tool was used to produce a parsimony tree. The consensus tree was estimated from equally parsimony trees with 100 boostraps and visualized by FigTree v.1.4.4 (https://github.com/rambaut/figtree/).

### Statistical analysis

Data analysis was performed using JMP Pro (version 16.0.0). Pearson’s χ^2^ test or Fisher’s exact test were used to compare the genotypes and virulence factors of infectious and non-infectious *S. aureus*, as well as identify factors involved in sepsis, ARDS, DIC, and bacteremia. Multivariate analysis was performed to identify factors associated with bacteremia, DIC, ARDS, and septic shock. A *P* value < 0.05 was considered statistically significant.

## SUPPLEMENTAL MATERIAL

Table S1 spectrum.02950-23-s0001.docx Baseline characteristic (All patients nasal swab performed).

Table S2 spectrum.02950-23-s0002.docx Factors associated with bacteremia, DIC, ARDS, septic shock.

Table S3 spectrum.02950-23-s0003.docx Prevalence of MRSA clones in this study.

ASM does not own the copyrights to Supplemental Material that may be linked to, or accessed through, an article. The authors have granted ASM a non-exclusive, world-wide license to publish the Supplemental Material files. Please contact the corresponding author directly for reuse.
