# Supplementary material for: Genomic characterization of Staphylococcus aureus isolated from patients admitted to intensive care units of a tertiary care hospital: epidemiological risk of nasal carriage of virulent clone during admission
Source: Microbiol Spectr. 2024 May 6;12(6):e02950-23. doi: 10.1128/spectrum.02950-23 (PMC11237438; doi:10.1128/spectrum.02950-23)
Supplement: Table S1 — Baseline characteristic (All patients nasal swab performed). [file spectrum.02950-23-s0001.docx]

Table S1. Baseline characteristic (All patients nasal swab performed)

| Characteristics | ALL |
| --- | --- |
|  | n=1,951 |
| Age, years, median (IQR) | 66 (46.0-77.0) |
| Sex, male, n (%) | 1,201 (61.6) |
| Comorbidity |  |
| Diabetes mellitus, n (%) | 399 (20.5) |
| Cardiovascular disease, n (%) | 473 (24.2) |
| Respiratory disease, n (%) | 176 (9.0) |
| Cerebrovascular disease, n (%) | 200 (10.2) |
| Cirrhosis, n (%) | 74 (3.8) |
| Renal failure, n(%) | 149 (7.6) |
| Solid cancer, n (%) | 304 (15.6) |
|  |  |
| Reason for admission |  |
| Endogenous disease, n (%) | 1,359 (69.7) |
| Infection, n (%) | 337 (17.3) |
| Heart disease, n (%) | 278 (14.2) |
| Neurological disease, n (%) | 220 (11.3) |
| Others, n (%) | 524 (26.9) |
| Exogenous disease, n (%) | 592 (30.3) |
| Trauma, n (%) | 370 (19.0) |
| Toxin, n (%) | 124 (6.4) |
| Others, n (%) | 98 (5.0) |
|  |  |
| SOFA score, median (IQR) | 4 (2.0-8.0) |
| APACHEII score, median (IQR) | 17 (11.0-25.0) |
| In-hospital mortality, n (%) | 299 (15.6) |
| ICU mortality, n (%) | 219 (11.2) |
