# Supplementary material for: Genomic characterization of Staphylococcus aureus isolated from patients admitted to intensive care units of a tertiary care hospital: epidemiological risk of nasal carriage of virulent clone during admission
Source: Microbiol Spectr. 2024 May 6;12(6):e02950-23. doi: 10.1128/spectrum.02950-23 (PMC11237438; doi:10.1128/spectrum.02950-23)
Supplement: Table S2 — Factors associated with bacteremia, DIC, ARDS, septic shock. [file spectrum.02950-23-s0002.docx]

Table S2. Factors associated with bacteremia, DIC, ARDS, septic shock. Multivariate analysis

| Variable | OR (95%CI) | *p* |
| --- | --- | --- |
| **Bacteremia** |  |  |
| Age | 1.00 (0.98-1.02) | 0.9947 |
| Sex, male | 0.33 (0.11-0.95) | 0.0399 |
| APACHEII score | 1.09 (1.03-1.15) | 0.0016 |
| *seb* | 7.80 (1.33-45.60) | 0.0226 |
|  |  |  |
| **ARDS** |  |  |
| Age | 1.02 (0.98-1.05) | 0.3626 |
| Sex, male | 0.24 (0.07-0.86) | 0.0286 |
| APACHEII score | 1.11 (1.04-1.19) | 0.0008 |
| *psm-mec* | 10.79 (1.96-59.22) | 0.0062 |
|  |  |  |
| **DIC** |  |  |
| Age | 0.99 (0.97-1.02) | 0.6443 |
| Sex, male | 0.82 (0.30-2.19) | 0.6882 |
| APACHEII score | 1.03 (0.98-1.08) | 0.2619 |
| *tst1* | 4.28 (1.33-13.73) | 0.0146 |
