# Supplementary material for: Genomic characterization of Staphylococcus aureus isolated from patients admitted to intensive care units of a tertiary care hospital: epidemiological risk of nasal carriage of virulent clone during admission
Source: Microbiol Spectr. 2024 May 6;12(6):e02950-23. doi: 10.1128/spectrum.02950-23 (PMC11237438; doi:10.1128/spectrum.02950-23)
Supplement: Table S3 — Prevalence of MRSA clones in this study. [file spectrum.02950-23-s0003.docx]

| Table S3. Prevalence of MRSA clones in this study. | |
| --- | --- |
| MRSA clones | number of strain (%) |
| Total MRSA | n=122 (100) |
|  |  |
| CC1: |  |
| ST1-MRSA-SCC*mec*IV | n=10 (8.2) |
| ST2725-MRSA-SCC*mec*IV | n=1 (0.8) |
|  |  |
| CC5: |  |
| ST5-MRSA-SCC*mec*II (New York/Japan) | n=8 (6.6) |
| ST764-MRSA-SCC*mec*II | n=14 (11.5) |
|  |  |
| CC8: |  |
| ST8-MRSA-SCC*mec*IVa-ACME (USA300) | n=0 (0) |
| ST8-MRSA-SCC*mec*IVl (MRSA/J) | n=19 (15.6) |
|  |  |
